# Formulations of Amlodipine: A Review

**DOI:** 10.1155/2016/8961621

**Published:** 2016-10-16

**Authors:** Muhammad Ali Sheraz, Syed Furqan Ahsan, Marium Fatima Khan, Sofia Ahmed, Iqbal Ahmad

**Affiliations:** Baqai Institute of Pharmaceutical Sciences, Baqai Medical University, 51 Deh Tor, Toll Plaza, Super Highway, Gadap Road, Karachi 74600, Pakistan

## Abstract

Amlodipine (AD) is a calcium channel blocker that is mainly used in the treatment of hypertension and angina. However, latest findings have revealed that its efficacy is not only limited to the treatment of cardiovascular diseases as it has shown to possess antioxidant activity and plays an important role in apoptosis. Therefore, it is also employed in the treatment of cerebrovascular stroke, neurodegenerative diseases, leukemia, breast cancer, and so forth either alone or in combination with other drugs. AD is a photosensitive drug and requires protection from light. A number of workers have tried to formulate various conventional and nonconventional dosage forms of AD. This review highlights all the formulations that have been developed to achieve maximum stability with the desired therapeutic action for the delivery of AD such as fast dissolving tablets, floating tablets, layered tablets, single-pill combinations, capsules, oral and transdermal films, suspensions, emulsions, mucoadhesive microspheres, gels, transdermal patches, and liposomal formulations.

## 1. Introduction

Amlodipine (AD) belongs to the group of calcium channel blockers. The newer calcium channel blockers such as dihydropyridines, AD, felodipine, and nisoldipine have improved vascular selectivity and longer durations of action. They bind to target receptors in a slow and sustained pattern producing a smooth onset of action with a 24 h control of blood pressure. Once daily dosing of these longer acting calcium channel blockers improves patient compliance and is associated with minimum encounter of side effects. The calcium channel blockers are suitable for a wide range of hypertensive patients including the elderly, black, and those with concomitant diseases that preclude the use of other antihypertensives [1].

AD is commonly used in the treatment of heart diseases like angina and hypertension [2]. Efforts have been made to prepare various dosage forms of AD to improve its efficacy and stability. Therefore, a comprehensive review of various formulations of AD reported in the literature has been made which would be helpful for pharmaceutical scientists and formulators in identifying and developing the most suitable dosage form of AD.

## 2. Formulations of AD

Formulations of AD may contain its different salts such as besylate, mesylate, or maleate. These salts are considered interchangeably and the strength of the dosage form is determined in terms of the parent molecule, that is, AD [2]. Amlodipine besylate (ADB) is the most commonly used derivative of AD which is used for the preparation of its various dosage forms. The salts of AD are known to affect the physicochemical properties of the drug as the besylate salt is known to have better water solubility than AD alone. Some newer salts of AD such as adipate [3], camsylate [4], and nicotinate [5] were developed and compared with ADB for their pharmacokinetics. It was found that all newer salts were bioequivalent and showed similar pharmacokinetic characteristics to those of ADB. ADB and AD camsylate are known to exert similar neuroprotective effects on primary cultured cortical neuronal cells by reducing oxidative stress, enhancing survival signals, and inhibiting death signals [6]. Different types of formulations of AD have been reported by various workers which are presented as follows.

### 2.1. Tablets

AD is commercially available in tablet dosage form in once daily doses of 5 and 10 mg. It is usually given orally in the besylate form. A 6.9 mg of ADB is equivalent to 5 mg of AD. It is well absorbed after oral administration and has a bioavailability of around 60–65% [7]. A racemate and* S*-enantiomer of ADB (levamlodipine besylate) were prepared and compared with each other. It was found that the two compounds have different melting points, solubility, dissolution rate, crystal morphology, particle size distribution, and hygroscopicity. The melting point of* S*-enantiomer was lower as compared to the racemate form which was due to its better water solubility and intrinsic dissolution property [8]. Furthermore, it has been reported that the calcium channel blocking activity of AD is restricted to its* S*-enantiomer, that is,* S*-AD [9, 10], while the other form, that is,* R*-AD exhibits a 1000-fold lower activity [10, 11]. It is interesting to note that AD is therapeutically used as a racemic mixture and both* S*- and* R*-forms are available in 1 : 1 ratio in conventional formulations. Therefore, a tablet formulation containing only* S*-form was compared with the conventional tablets of AD. Both formulations were found to be bioequivalent and well tolerated and no difference was observed in terms of pharmacodynamic profiles and clinical effects. Both formulations were found to be well tolerated and no serious adverse effects were noted [10]. Similarly, a tablet formulation of* S*-enantiomer of ADB was prepared with compatible excipients. The formulation was optimized using full factorial design and was found within limits when compared to the commercial brands of ADB in terms of appearance, dissolution, and stability [8]. On the contrary, an in vitro study was performed to evaluate the ability of AD and its enantiomers (*S*- and* R*-) to release nitric oxide in isolated coronary microvessels and to regulate tissue oxygen consumption via nitric oxide release. It was found that AD and its* R*-form released nitric oxide in a concentration dependent manner and reduced oxygen consumption in canine cardiac tissues while no effect was observed by the* S*-form at any concentration in either case [11].

Since AD is a photosensitive drug, an attempt has been made to formulate tablets by liquisolid technique in order to provide photoprotection to the active drug. The tablets were prepared using propylene glycol as solvent in 1 : 1 ratio with AD, avicel PH102 as carrier, amorphous silicon (nanometer size), and titanium dioxide either alone or in combination as coating agents. These tablets were exposed to different radiations such as visible, UVA, and UVB for 8 h to determine the photoprotective effect of the coating material. The results indicated that liquisolid technique not only provided photoprotection but also improved the dissolution rate of AD. Therefore, this technique can be used as an alternative to conventional coating methods particularly in formulations containing photosensitive drugs [12].

#### 2.1.1. Fast Dissolving/Orodispersible Tablets

In addition to the presently marketed AD tablets and capsules, the novel fast dissolving tablets (also known as orodispersible, fast melting, fast dispersing, rapid dissolve, rapid melt, and/or quick disintegrating tablet formulations) offer an option to the patients who have difficulty in swallowing (i.e., dysphagia). It is a convenient and modest method of administration without the need of water [13, 14] with good palatability [15, 16]. The dispersible tablets of ADB are comparatively bioequivalent to conventional tablets [17]. The main drawback of using orally disintegrating or crushed tablets of AD is their bitter taste [16, 18, 19] and, therefore, taste masking of such dosage forms is very important to increase palatability [15].

The fast dissolving oral ADB tablets were prepared by direct compression method using croscarmellose (Ac-Di-Sol), crospovidone (Kollidon CL), and sodium starch glycolate as superdisintegrants in varying concentrations of 2, 4, and 6%. Among the three disintegrants, croscarmellose in 6% concentration was found to be the most rapid by causing disintegration of the tablets within 11 s [20]. On the contrary, sodium starch glycolate and croscarmellose showed comparatively better results among the three disintegrants when used in the concentrations of 5–10% [21]. In another study, fast dissolving tablets of ADB were prepared by incorporating crospovidone and sodium starch glycolate either alone or in combination. The tablets prepared using a combination of both superdisintegrants were found to have most rapid disintegration and highest dissolution rate as compared to formulations containing a single superdisintegrants [22]. Therefore, it has been recommended to use a combination of superdisintegrants for the formulation of fast dissolving tablets of ADB [22, 23]. The solubility and dissolution of ADB was reported to increase when its solid dispersion was prepared with crospovidone by solvent evaporation method and then the mouth dissolving tablets were manufactured in 1 : 4 ratio [24]. The orodispersible tablets of AD are known to provide equivalent systemic effect to that of the conventional AD tablets or capsules when administered with or without water [25].

The dissolution of ADB was also enhanced when* Plantago ovata* mucilage (Ispaghol) was used as a natural superdisintegrant. The fast dissolving tablets were prepared by direct compression method and the concentration of the mucilage was found to be a key factor in affecting the rate of disintegration because a decrease in time with an increase in the concentration of the superdisintegrant has been noted [26]. Similarly, fenugreek seed mucilage and* Ocimum basilicum* (basil) gum were also employed as natural superdisintegrants in the formulation of fast dissolving tablets of ADB. The tablets were prepared by direct compression method using either of the superdisintegrants in varying concentrations (i.e., 2–10 mg). It was observed that the formulations containing the highest amount of the disintegrant showed the fastest disintegration due to rapid wetting, dispersion, and water-absorption ratio. Moreover, the tablets were also found stable for three months when stored at 25 ± 20°C/60 ± 5% RH and 40 ± 20°C/75 ± 5% RH [27].

#### 2.1.2. Sustained Release Floating Tablets

Floating drug delivery systems or hydrodynamically balanced systems are those that have lower bulk density than the gastric fluids. Due to this property the tablet remains buoyant for prolonged period of time and thus releases the drug slowly at a desired rate. However, weight measurements and swelling properties are other two important parameters required for the buoyant capabilities of the tablets. Polymers play an important role in controlling the release of the drug in such systems. Sometimes an effervescent component is also added that liberates CO_2_ when it comes in contact with the acidic gastric fluids. The liberated gas is entrapped in the gellified hydrocolloid and produces an upward motion causing floating of the tablet [28].

Pare et al. [29] formulated various floating effervescent tablets of ADB and studied their different physicochemical properties including the release of drug from its dosage form. The tablets were prepared by direct compression method using different hydrophilic (hydroxypropyl methylcellulose) and hydrophobic (Carbopol 934P) polymers and effervescing agents such as sodium bicarbonate and citric acid. The formulated tablets were compared with the commercial dosage forms and it was observed that their release was twice (i.e., 24 h) delayed to those of the marketed products (i.e., 12 h) [29].

#### 2.1.3. Layered Tablets

Formulation of layered tablets has gained considerable popularity due to their certain advantages over conventional monolayer tablets. These advantages may include the use of two active drugs at the same time, formulation of two chemically incompatible substances in bilayer form, increased efficacy in case of combining two drugs with synergistic effect, use of two different release profile forms (quick and delayed release) of a single active drug, and better patient compliance due to decreased dosing burden [30, 31].

Layered tablets of ADB and atenolol were prepared as either monolayer (mixed matrix) or bilayer using similar excipients and processing technique. The bilayered tablet formulation was found to be more stable than the monolayered type. The packaging material also affected the stability of the formulations and it was observed that the stability of the tablets was increased when packed in aluminium strips compared to PVC blisters. In either case degradation in ADB was prominent whereas no decline in content was noted for atenolol [32]. In another study, an attempt was made to prepare bilayered effervescent floating tablets of ADB and hydrochlorothiazide by direct compression using various concentrations of polymers (various grades of hydroxypropyl methylcellulose such as K4M, K15M, K100M, and polyvinylpyrrolidone PVP K30). The tablets were formulated in a way to release ADB immediately in gas layer and hydrochlorothiazide with a sustained action. ADB was released more than 85% within 15 min while hydrochlorothiazide was released over a period of around 8 h [33]. Similarly, a bilayer combination of ADB with metoprolol succinate has also been reported with immediate and sustained release of each drug. The tablets were prepared by direct compression using sodium starch glycolate and pregelatinized starch as superdisintegrants and hydroxypropyl methylcellulose (K4M and K100) for sustained action. The tablets showed a first-order release of about 98% for ADB at 30 min and around 90% for metoprolol succinate in 20 h. The stability studies performed for blister packed tablets at 25°C/60% RH and 40°C/75% RH for three months were also found satisfactory [34].

#### 2.1.4. Single-Pill (Fixed-Dose) Combinations

Film coated immediate release tablets of the combination of valsartan and AD were formulated and evaluated for their in vivo release profile and stability. The core tablets of the combination were prepared by dry granulation and the film coating was performed using Opadry aqueous coating dispersion. It was found that the release of each drug from the combination tablet meets the FDA guidelines for bioequivalence studies. The stability of the formulated tablets was evaluated according to the guideline of ICH. The film coated tablets were found stable both on long term (12 months) and accelerated stability studies [35]. Similarly, a study was performed to compare the pharmacokinetic profile of a combination of AD (5 mg) and benazepril (10 mg) in tablets and capsules. The results indicated both dosage forms containing the combination of actives to be bioequivalent and well tolerated [36].

A large number of pills for the treatment of a single or multiple diseases are a major source of noncompliance in patients. Therefore, a single-pill or fixed-dose combination therapy is often preferred to reduce the polypharmacy burden and improve patients' compliance [37, 38]. A number of workers have employed AD in combination with other drugs as a single-pill and used it clinically with reasonable success. Not much information regarding the formulations is available as the studies are more clinically oriented and the combinations have been discussed with respect to their clinical outcomes only in majority of the cases. The drugs most commonly employed in combination with AD (i.e., single drug + AD combination) include aliskiren [39–41], atorvastatin [42–52], azilsartan [53], benazepril [54, 55], fimasartan [56], indapamide [57, 58], irbesartan [59], olmesartan (olmesartan medoxomil) [39, 60–67], perindopril [68–72], telmisartan [73–77], and valsartan [70, 71, 78–97].

Some workers have tried triple combinations in a single pill and used it clinically, that is, two different drugs + AD. The drug combinations with AD include aliskiren and hydrochlorothiazide [98, 99], olmesartan and hydrochlorothiazide [67, 100–102], and valsartan and hydrochlorothiazide [88, 95, 101–103].

### 2.2. Capsules

Solid dispersion of drugs may provide fast dissolution but requires some time to initiate its release. For the drugs used in emergency conditions, like antihypertensive drugs, a lag time of almost zero is desirable. In order to reduce the lag time in drug release and enhance the dissolution, semisolid matrix-filled hard gelatin capsules of ADB were prepared by Tyagi et al. [104]. The solid dispersions of ADB were prepared through fusion method using varying concentrations of Poloxamer 407 and Plasdone S630. The solid dispersion that showed better dissolution profile was selected for the preparation of semisolid matrix. The optimized solid dispersion of ADB was mixed with Gelucire 44/14 and polyethylene glycol (PEG) 400 to make it semisolid and further mixed with PEG 6000 to suspend the particles in the matrix. The suspended particles were then transferred into hard gelatin capsules. Making semisolid matrix resulted in a threefold decrease in the lag time of ADB release as compared to its amorphous solid dispersions indicating that this approach can be used for the rapid therapeutic effect of the drug. The accelerated stability studies of these capsules revealed that the preparations were stable for at least three months when stored at 40 ± 2°C and 75 ± 5% RH [104].

Asymmetric membrane capsules for simultaneous delivery of ADB and atenolol were formulated by dip coating (wet phase inversion) method using cellulose acetate as polymer in 10% concentration. Each active ingredient was mixed separately with the osmotic agents, that is, potassium chloride and citric acid monohydrate. Atenolol (50 mg) was filled manually in the body while ADB (5 mg) in the cap. Compartments in the capsules were formed by using paraffin wax plug. A layer of the polymer was applied over it to avoid any leakage from each compartment followed by outer sealing of the capsules with a solution (10% w/v cellulose acetate in a mixture of acetone and alcohol). The release data showed around 55% release of ADB at 12 h from capsules containing no osmotic agent while a 100% release was noted at 6 h for capsules containing 35 mg of citric acid and 20 mg of potassium chloride. In the latter case, the caps of the capsules were modified by increasing concentration of the polymer to 15%, which resulted in a sustained release of ADB (~95%) for 13 h indicating the role of the polymer in slowing down the disintegration of the capsules cap. A zero-order release was observed in the majority of cases. All capsules were found to be stable for three months when stored either in sealed or unsealed containers at 40 ± 2.0°C/75 ± 5% RH [105].

### 2.3. Oral Films

The in vitro disintegration studies for fast disintegrating films of ADB have been conducted by Shelke et al. [106] through the solvent casting technique. Sodium alginate was used as a film polymer and sodium starch glycolate as disintegrant in nine different concentrations. The study was carried out at pH 6.2 and it was observed that the sodium alginate film on the tablet started to disintegrate in 30 s and almost 75–80% of the drug was released after 6 min in all the formulations [106].

### 2.4. Suspensions

In addition to many other formulations the extemporaneous suspensions of ADB (1 mg/mL) have also been prepared from the commercially available oral tablets. One of the suspensions was made by using methylcellulose (1%) in syrup and the other using a 1 : 1 mixture of commercially available extemporaneous syrup vehicles, Ora-Plus and Ora-Sweet. The suspensions were stored in plastic bottles at room (25°C) and refrigerated (5°C) temperatures and were analyzed for their physical and chemical stability for a period of three months. The results revealed that suspensions kept under refrigerator were more stable physically and chemically to those stored at room temperature. The assay results indicated that the suspensions stored in a refrigerator retained >90% of the drug for 91 days compared to only 56 days for the same concentration in the samples stored at room temperature. Moreover, a slight variation in pH was also observed in the suspensions containing methylcellulose and kept at room temperature. On the basis of these findings, it was suggested to use these suspensions as per body weight in pediatrics as well as in elderly patients who have difficulty in swallowing [107]. No significant difference in bioavailability has been reported between suspensions prepared from crushed tablets and oral tablets of AD when studied in healthy volunteers. However, the suspension was found to be very unpalatable [108].

A similar suspension formulation has also been used successfully by Rivero et al. [109] in the treatment of a 5 year old girl who developed hypertension due to a consequence of other complications. She was initially treated with nifedipine and captopril but due to poor prognosis was shifted to AD and enalapril, each in an oral dose of 2.5 mg/day that showed better therapeutic effect. The tablets for adults are available in a dose of 5 and 10 mg, therefore, the prescribed dose was administered in the form of a suspension in a similar dose of 1 mg/mL as reported by Nahata et al. [107] using a mixture of Ora-Plus and Ora-Sweet [109].

### 2.5. Nanoemulsions

Targeted drug delivery system is gaining popularity day by day which has encouraged researchers towards the development of nanopreparations. Various oil-in-water nanoemulsions of AD were prepared by using different concentrations of oleic acid (oil phase), tween 20 (surfactant), Transcutol P (cosurfactant), and constructing pseudoternary phase diagrams. The transdermal effect of nanoemulsions on rat's skin was determined using Franz diffusion cell. The optimum concentration of excipients that showed highest rate of permeation in the formulation was 2% for oil, 20% for surfactant, and 10% for cosurfactant. The rate of permeation was found to be affected inversely with the excipient concentrations as the highest rate was achieved with the lowest oil and surfactants concentrations. The results showed a positive response in transdermal drug delivery and proved that the nanoemulsion is a potential vehicle for enhancing skin permeation of AD through transdermal preparations [110].

Similarly, oral nanoemulsions of ADB were prepared by spontaneous emulsification method with the purpose of obtaining increased bioavailability and targeted drug delivery to the site of action. Partition coefficient, droplet size, and in vitro release of drug were analyzed in nanoemulsions following construction of pseudoternary phase diagrams. The release of ADB from oral nanoemulsions was found to be much higher than those of the tablets available in the market. The oral administration of radiolabeled formulations (^99m^Tc-labeled) in mice indicated about three times higher efficacy of nanoemulsions as compared to ADB suspensions. The higher bioavailability and biodistribution of ADB from nanoemulsions were due to better uptake of the drug in all organs, thus indicating the advantage of using nanoemulsions as carriers for the delivery of AD [111].

Another novel approach employed to enhance the bioavailability and photostability of AD was the preparation of redispersible dry emulsions by spray drying technique as oil-in-water emulsions. The redispersion of the dried emulsion in distilled water formed the emulsion but with a larger particle size which was 1.4-fold higher than the original emulsion (i.e., 0.24 ± 0.30 *μ* versus 0.17 ± 0.02 *μ*). However, no further change in the droplet size of dry emulsions was observed during six months of storage at room temperature. The dried emulsions showed a significant photoprotection of AD as only 6% of the drug was degraded in comparison to 70% in ethanolic solution and 23% as dry powder when exposed to UV irradiation for 12 h. Similarly, the in vitro release of dried emulsions (66%) was also found to be higher than that of the powder form (48%) at 60 min time interval with a 2.6–2.9-fold higher bioavailability as observed in rats after oral administration. All these results indicated that the dried emulsions of AD could be used as an effective alternative drug delivery system [112].

### 2.6. Mucoadhesive Microspheres for Nasal Delivery

Local or systemic delivery of drugs through nasal route has been used conventionally for the treatment of different local diseases. In the recent past, this route has gained considerable popularity because of its reliability for the delivery of drugs that are ineffective from oral route due to metabolism in the gastrointestinal tract [113, 114]. The recent developments and the factors affecting nasal drug delivery systems have been reviewed by Mainardes et al. [113] and Jadhav et al. [114]. Nasal delivery of AD as mucoadhesive microspheres is another field of interest for pharmaceutical scientists as some interesting progress has been made in this regard. AD loaded microspheres are known to provide highest photostability as compared to other dosage forms including ethanolic solutions, powder, tablets, liposomes, and cyclodextrin complexes [115].

A study has been performed in which mucoadhesive chitosan microspheres of ADB were prepared by simple emulsification cross-linking using glutaraldehyde as cross-linking agent. Various formulation parameters such as drug-polymer ratio (1 : 1, 1 : 2, 1 : 3, 1 : 4, and 1 : 5), stabilizing agent concentration (0.1, 0.2, and 0.3% w/v), and stirring rate (600 and 1200 rpm) were studied. It was observed that the in vitro mucoadhesive property and size of the microspheres increased from 28 *μ* to 52 *μ* with an increase in chitosan concentration which could be due to the amino groups available for binding with the sialic acid residues in mucus layer and cross-linking of the polymer. On the contrary, the release of ADB was slowed down with an increase in polymer concentration from about 85% to 76% after 8 h due to matrix formation. Increase in the stirring rate reduced the median particle size diameter of the microspheres from ~99 *μ* to 36 *μ*. The microspheres were found to be spherical with smooth surface without any aggregation and were, therefore, suggested as suitable for nasal administration [116].

Similarly, hydroxypropyl guar (HPG) microspheres containing ADB were formulated for nasal administration by water-in-oil emulsification solvent evaporation technique in order to investigate their suitability as nasal drug delivery system. The formulation variables which could affect the preparation of the microspheres including drug-polymer (0.5 : 3, 1 : 3, 1.5 : 3, and 2 : 3), polymer-drug (1 : 1, 2 : 1, 3 : 1, and 4 : 1), and emulsifier (0.2, 0.3, 0.4, and 0.5% w/v) concentrations, temperature (60, 70, 80, and 90°C), and agitation speed (1400, 1600, 1800, and 2000 rpm) were also studied. It was observed that an increase in emulsifier concentration, temperature, and agitation speed resulted in a decrease of particle size from about 132 *μ* to 19 *μ*. The microspheres were found to be spherical and free flowing and showed good mucoadhesive and swelling properties. ADB was released from microspheres with a sustained action of over 8 h. The drug entrapment efficiency of the microspheres was found to be affected by drug and polymer concentrations. An increase in the HPG concentration resulted in higher ADB entrapment with an increase in the size of the microspheres. Similarly, an increase in the drug concentration also resulted in an increased particle size but the drug entrapment was found to decrease. The formulations were also found to be stable at room (25 ± 2°C/60 ± 5% RH), refrigeration (5 ± 3°C), and accelerated (40 ± 2°C/75 ± 5% RH) temperatures. No significant changes were observed at the end of 90 days indicating the suitability of ADB loaded microspheres for nasal administration [117].

### 2.7. Topical Formulations

#### 2.7.1. Gels

Gel formulations containing dexamethasone (0.3%) and ADB (0.5%) have been formulated using carboxymethyl cellulose sodium as gelling agent, azone (laurocapram) as penetration enhancer, and propylene glycol as solvent and humectant. The gels were studied for drug penetration within flap tissues through excised rat skin. It was observed that the compound gel containing both drugs can penetrate into skin tissue which could significantly increase the survival of ischemic skin flap [118]. The gels of ADB have also been used for the treatment of feline hypertension (i.e., in cats). Gels were prepared in a commercially available vehicle, Lipoderm, which contains a proprietary liposomal component and has a smooth creamy texture and does not separate on refrigeration [119].

ADB loaded nanostructured lipid carrier gels or nanogels for intranasal delivery have been prepared by emulsion solvent diffusion and evaporation method followed by ultrasonication. Various properties of the nanogels including zeta potential, particle size, and entrapment efficiency were investigated. The gels were also characterized for drug content, pH, and in vitro and ex vivo drug diffusion. All these studies showed that the nanogels of ADB have good sustained action as compared to the pure drug solution and nanostructured lipid carrier dispersion and, therefore, can be used for intranasal delivery of ADB [120].

#### 2.7.2. Transdermal Patches and Films

Drug-in-adhesive transdermal patches have been prepared using* S*-amlodipine (*S*-AD) free base (i.e., levamlodipine) and studied for in vitro and in vivo activity in rats after optimization of the formulation parameters. The* S*-AD (4%) loaded patches were prepared using appropriate amounts of pressure-sensitive adhesive. Penetration enhancers were added to acetic ether followed by agitation at room temperature and then applied to the surface of a fluoropolymer-treated polyester release liner (ScotchPak® 1022). The mixture was than subjected to drying, cutting, and covering with a polyester backing membrane (ScotchPak 9726) followed by storage in an aluminium-plastic membrane. The patches were applied transdermally to the rats and the plasma level of* S*-AD was measured at different time intervals. It was observed that the patches can maintain drug plasma level for up to 72 h without any first pass effect and hence can be used as an effective alternative dosage form in the treatment of hypertension [121].

In another study, an attempt was made to formulate various ADB (10%) loaded films by solvent evaporation technique using different polymers such as polycaprolactone, ethylcellulose, polylactic acid, and polyhydroxyethyl methacrylate. Polyethylene glycol 400 was used as a plasticizer in a 2% concentration in each formulation. The prepared films were evaluated for parameters such as weight variation, thickness, drug content, and in vitro permeation. All films showed good physicochemical performance. The in vitro release studies indicated sustained release of ADB from all films over a period of 24 h with the slowest rate in films prepared from polyhydroxyethyl methacrylate (i.e., 79%) [122].

### 2.8. Liposomal Formulations

Liposomes are spherical vesicles with at least a single lipid bilayer that are used to deliver a drug to a specific site or target in the body and to overcome any stability or toxicity related problems of the drug. Liposomes have gained tremendous popularity due to their ability of delivering both lipophilic and hydrophilic drugs [115]. AD is a lipophilic drug and is known to have better penetration ability into liposomal vesicles than other cardiovascular agents such as mexiletine and indapamide [123].

Since AD is sensitive to light, it requires protection to avoid degradation. To overcome this problem, a liposomal preparation of AD was prepared and evaluated for its photostability. It was observed that an inclusion complex between the liposome and drug was formed that resulted in a significant reduction in the photodegradation of AD which was higher than some other systems like solution and powder. The degradation time of 10% drug (*t*_0.1_) was found to be about 220 min which was better than the ethanolic solutions (*t*_0.1_ = ~10 min) and powder form of AD (*t*_0.1_ = 111 min) [115].

AD possesses antioxidant properties and plays an important role in the apoptosis [124–129]. Due to this activity, targeted delivery of AD in liposomal preparations, either alone or in combination with other drugs, has been employed by various workers with significant success [124, 125, 129].

## 3. Conclusion

For an effective treatment, it is of utmost importance to deliver the drug in a suitable dosage form through an appropriate route. A dosage form must be designed in a way that not only provides stability to the formulation ingredients but also gives optimum therapeutic effect. Different formulations of AD have been developed and the majority of these have shown acceptable stability. However, further studies regarding their in vivo efficacy and more innovations in AD delivery systems would definitely help in the effective treatment of various diseases. Availability of different dosage forms of AD would also provide a choice to physicians and pharmacists to select the most appropriate delivery system based on the patient's condition.
